# Impact of COVID‐19 on Time to Treat Breast Cancer and Racial Disparities Among Women in the Military Health System From FY2018‐2022

**DOI:** 10.1002/cam4.71292

**Published:** 2025-10-06

**Authors:** Melody F. Mullin, Amanda Banaag, Christian Coles, Yvonee Eaglehouse, Kangmin Zhu, Tracey P. Koehlmoos

**Affiliations:** ^1^ Department of Preventive Medicine and Biostatistics Uniformed Services of the Health Sciences Bethesda Maryland USA; ^2^ Center for Health Services Research Uniformed Services of the Health Sciences Bethesda Maryland USA; ^3^ Henry M. Jackson Foundation for the Advancement of Military Medicine, Inc. Bethesda Maryland USA; ^4^ Murtha Cancer Center Research Program, Department of Surgery Uniformed Services University of the Health Sciences Bethesda Maryland USA

**Keywords:** breast cancer, COVID‐19 pandemic, equal access to healthcare, military health system, timely treatment

## Abstract

**Background:**

Breast cancer is the most diagnosed cancer among women in the United States, and early identification and initiation of treatment are critical to improving outcomes. This study aims to investigate the breast cancer time to treat trends among women in the Military Health System before and during the COVID‐19 pandemic and if racial or socioeconomic disparities existed in timely treatment.

**Methods:**

A retrospective cohort study of all female MHS beneficiaries ages 18–63 years during fiscal years 2018–2022. Incident breast cancer was defined as one inpatient or three outpatient diagnoses of breast cancer within a 90‐day period and no previous breast cancer diagnosis in the 3 years prior. Time to treatment was calculated in days and timely treatment was identified if received within 90 days (surgical intervention) or 120 days (chemotherapy and radiation) after initial diagnosis. Study analyses included a *t*‐test and Kaplan–Meier curve for time to treatment and an adjusted modified Poisson regression for the relative risk of timely treatment.

**Results:**

A cohort of 14,286 women with incident breast cancer was identified; 94% received timely treatment. The average time to treatment was greater during the pandemic period (47.7 days, 95% CI = 46.6–48.7) compared to the pre‐pandemic period (44.8 days, 95% CI = 43.7–45.9). Regression results indicated no difference in the likelihood of timely treatment in the pandemic period (0.99 aRR, 0.98–1.01 95% CI), no racial or socioeconomic disparities, and timely treatment was more likely to be received in the direct care setting (aRR = 1.04, 95% CI = 1.01–1.07).

**Conclusion:**

Despite facing access to care challenges compounded by the COVID‐19 pandemic, the MHS was able to provide timely treatment to women for incident breast cancer. In addition, this study observed no racial or socioeconomic disparities in the timely treatment of breast cancer in a population with equal access to care.

## 1 Introduction

Breast cancer is the most prevalent diagnosed cancer among women in the United States and the second leading cause of cancer death [1]. In 2022, there were approximately 4.1 million women with a history of breast cancer in the United States. The American Cancer Society reported that one in eight women will receive a diagnosis of breast cancer in 2022 and 1 in 39 women will die, equating to over 43,000 women [2]. Early identification and initiation of treatment following a diagnosis of breast cancer are critical to improving outcomes [3], and time to treatment is a frequently used healthcare quality metric in breast cancer care [4, 5]. The suggested timeline between diagnosis and treatment is less than 90 days for surgical intervention, < 120 days for chemotherapy, and within 365 days for radiation therapy post chemotherapy [6].

It is well known that Black women have the lowest 5‐year relative survival of any racial/ethnic group for every molecular subtype and stage of disease (except Stage I) [2]. Racial disparities are hypothesized to be due to inadequate access to care and insurance status; however, research has found disparities continue to exist even in comprehensive healthcare systems [7]. These pre‐existing challenges are exacerbated by the COVID‐19 pandemic by restricting in‐person evaluations and massive redistribution of healthcare resources. This redirection of resources has led to delays and cancelations of nonurgent procedures and services. Across the board, there was a significant decrease in preventive screenings and early diagnosis of chronic conditions [8].

The Military Health System (MHS) is a comprehensive health system providing care to the Department of Defense (DOD) beneficiary population, which is inclusive of active duty service members, activated National Guard, retirees, and their eligible dependents. The MHS provides health care through means of direct care, meaning treatment is conducted within a military healthcare facility, or through the private care sector as an insurance benefit of TRICARE. The military treatment facility acts as the primary care manager (PCM) who then works directly with specialty facilities within the MHS to provide advanced care after referral. Most treatment is then conducted within the specialty military hospital; if the treatment or specialty is unavailable within the MHS, it will then be referred into the private care sector and covered by TRICARE. The MHS offers comprehensive care, at very little to no cost, to all beneficiaries, giving theoretical equal access to care without financial hardship. However, recent studies of the MHS found continued racial‐ethnic [9] and socioeconomic disparities in the time to treat for newly diagnosed breast cancer cases [10]. Furthermore, Mani et al. [11] concluded that race and socioeconomic factors were associated with decreased breast cancer screening rates in the MHS during the COVID‐19 pandemic [11].

Given the previous findings on the impact of the COVID‐19 pandemic on breast cancer screening rates and the comprehensive access to health care in the MHS, this study aimed to, (1) evaluate the impact of the COVID‐19 pandemic on time to treatment and timely treatment in female breast cancer patients and, (2) determine if any racial‐ethnic and socioeconomic disparities are associated with the timely initiation of treatment for incident breast cancer. We hypothesized that the time to treatment initiation for incident breast cancer was negatively affected by the COVID‐19 pandemic and that racial‐ethnic disparities in timely initiation of treatment were present within the MHS.

## 2 Methods

### 2.1 Study Design and Data Collection

**FIGURE 1:**
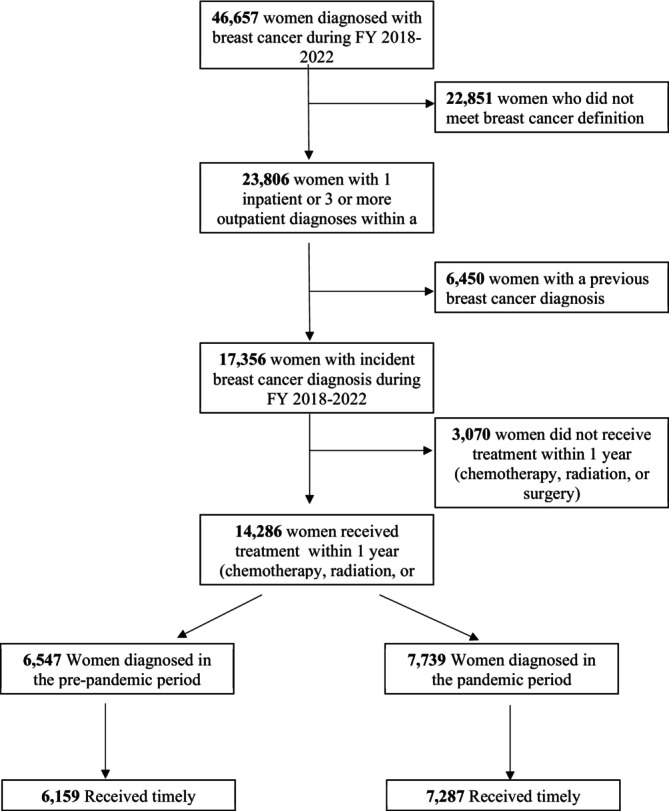
CONSORT diagram of the study cohort selection and distribution of the final cohort by COVID‐19 pandemic study periods.

We performed a retrospective cohort study on all female MHS beneficiaries, ages 18–63 years, who were diagnosed with breast cancer between fiscal years (FY) 2018–2022 (October 1, 2017—September 30, 2022). Demographic and clinical data for the study cohort during the study period were obtained from the MHS Data Repository (MDR), a central source of health care and pharmaceutical data delivered in the MHS for all beneficiaries [12]. The MDR does not include care from the Veterans Affairs health care system or health care delivered in theater (warfare). Patients ages 64 and older were excluded due to Medicare being the primary payer at age 65, which results in a loss of care transparency, and to account for the follow‐up time for timely treatment after initial diagnosis. Additionally, women who did not meet the incident breast cancer definition and who did not receive treatment in the MHS within 1 year after incident diagnosis were excluded from the study. Figure 1 details the identification process of the included cohort.

In order to identify patients with incident breast cancer diagnosis, this study utilized the incident cancer definition published in the Armed Forces Health Surveillance's Medical Surveillance Reporting System by [13]. Incident breast cancer was defined as one inpatient diagnosis or three unique outpatient diagnoses of breast cancer within 90 days and no previous diagnoses of breast cancer in the previous 3 years. International Classification of Diseases, 9th and 10th revision, (ICD‐9/ICD‐10) codes were used to identify breast cancer diagnosis, to include: ICD‐9 codes 174.0–174.9 and 233.0, and ICD‐10 codes C50.0‐C50.9 and D05.0‐D05.9. The cohort was followed for 1 year after initial diagnosis and first line of treatment (chemotherapy, radiation, or surgery) was identified through the use of Common Procedure Terminology (CPT) and ICD‐10‐CM and ‐PCS codes. A full list of codes used to identify treatment can be found in supplemental materials. Treatment was considered timely if it was received within 90 days after the first date of diagnosis for surgical intervention, or within 120 days after the first date of diagnosis for chemotherapy or radiation [6]. Patient demographics such as age, race or ethnicity (recorded in the MDR as American Indian/Alaskan Native, Asian/Pacific Islander, Black, Hispanic, White, and Other), beneficiary status, and rank or sponsor's rank (categorized as Junior Enlisted, Senior Enlisted, Junior Officer, Senior Officer, Warrant Officer, and Other) were retained from their health care record at the time of incident breast cancer diagnosis and used in analysis. ‘Other’ race is a self‐selected option available to those that do not identify with any of the available racial groups and could include those identifying as multiracial. ‘Other’ rank could include cadets, government personnel with access to the MHS, and anyone with incomplete but not missing rank details. The period in which the incident diagnosis occurred in relation to the start of the COVID‐19 pandemic was also recorded and used in analysis. The pre‐pandemic period was defined as October 2017–February 28, 2020, and the pandemic period was defined as March 1, 2020–September 30, 2022.

### 2.2 Study Analyses

The primary outcome of interest was time to treatment and was analyzed in two forms: (1) as a continuous variable recorded as the number of days between diagnosis and the first line of treatment within one year following diagnosis, and (2) as a dichotomous variable indicating if treatment was timely (within 90 days for surgery or 120 days for chemotherapy or radiation). Study analyses included descriptive statistics on patient demographics, unadjusted t‐test to assess for differences in time to treatment by pandemic period of diagnosis, Kaplan–Meier survival curve analysis to compare the mean time to treatment between pandemic periods, and unadjusted and adjusted modified Poisson regressions. A modified Poisson regression, with 95% confidence intervals and adjusted by age and beneficiary status, for risk ratios (aRR) was used to assess for the likelihood of timely treatment by pandemic period of diagnosis, race, rank, and care setting of treatment. Due to approximately 50% of the cohort having a missing race, the Reweighted Estimated Equations method for missing data [14] was used to impute missing race. All patient demographics, period of diagnosis, and timely treatment status were used in the reweighting calculations of an observed race. Subset analyses with modified Poisson regression models were performed to determine if significant interactions exist between pandemic periods and race/ethnicity and between pandemic periods and care setting of treatment. There is the potential for timely treatment to be affected differently in patients diagnosed in the few months prior to the U.S. declaring COVID‐19 a public health emergency compared to those diagnosed in 2019. To account for this potential issue, a modified Poisson regression model with patients diagnosed between January and March 2020 excluded was performed and no differences were observed in the results when compared to regression analyses with these patients included (Table S1). All analyses were performed using SAS, version 9.4 and statistical significance was set to *p*‐value < 0.05. This study protocol was reviewed by the Uniformed Services University Institutional Review Board and received an exempt determination under the provision of DoD regulation 32 CFR 219.104(d)(4) and an approved waiver from obtaining informed consent under DOD 6025.18‐R, C7.9.2.2.

## 3 Results

We identified and screened 46,657 women in the MHS diagnosed with breast cancer during FY 2018–2022; after exclusions were applied, 14,286 women remained eligible for inclusion in our study cohort (Figure 1).

Table 1 details the distribution of cohort demographics and the period of diagnosis by timely treatment status. The average age at diagnosis of women in the study cohort was 52.30 years (8.73 SD) and the majority of women were White (27.40%), dependent beneficiaries (82.22%), associated with a Senior Enlisted rank (67.86%), diagnosed in the pandemic period (54.17%), received treatment in the private sector (81.04%), and received timely treatment (94.12%).

**TABLE 1:** Characteristics of incident breast cancer cohort.

	Delayed treatment	Timely treatment	Total cohort
**Total (% of total cohort)**	840 (5.88)	13,446 (94.12)	14,286 (100)
	***n* (%)**	***n* (%)**	***n* (%)**
**Mean age (SD)**	51.86 (8.49)	52.33 (8.74)	52.30 (8.73)
**Race**			
White	231 (27.50)	3683 (27.39)	3914 (27.40)
Black	93 (11.07)	1343 (9.99)	1436 (10.05)
Hispanic	41 (4.88)	525 (3.90)	566 (3.96)
Asian/Pacific Islander	27 (3.21)	441 (3.28)	468 (3.28)
American Indian/Alaskan Native	< 11	< 11	45 (0.31)
Other	48 (5.71)	715 (5.32)	763 (5.34)
Missing	397 (47.26)	6697 (49.81)	7094 (49.66)
**Beneficiary status**			
Active duty	23 (2.03)	278 (2.07)	301 (2.11)
Dependent of guard/reserve	47 (5.60)	814 (6.05)	861 (6.03)
Dependent	682 (81.19)	11,064 (82.28)	11,746 (82.22)
Guard/reserve	13 (1.55)	213 (1.58)	226 (1.58)
Retiree	69 (8.21)	965 (7.18)	1034 (7.24)
Missing	< 11	< 11	118 (0.83)
**Rank**			
Junior enlisted	31 (3.69)	363 (2.70)	394 (2.76)
Senior enlisted	564 (67.14)	9130 (67.90)	9694 (67.86)
Junior officer	88 (10.48)	1336 (9.94)	1424 (9.97)
Senior officer	125 (14.88)	2072 (15.41)	2197 (15.38)
Warrant officer	27 (3.21)	463 (3.44)	490 (3.43)
Other	< 11	< 11	44 (0.31)
Missing	< 11	< 11	43 (0.30)
**Period of diagnosis**			
Pre‐pandemic	388 (46.19)	6159 (45.81)	6547 (45.83)
Pandemic	452 (53.81)	7287 (54.19)	7739 (54.17)
**Care setting of treatment**			
Direct care	134 (15.95)	2575 (19.15)	2709 (18.96)
Private sector care	706 (84.05)	10,871 (80.85)	11,577 (81.04)

*Note:* Cell counts of 10 or fewer were censored to protect the anonymity of patients and to prevent back calculation.

**FIGURE 2:**
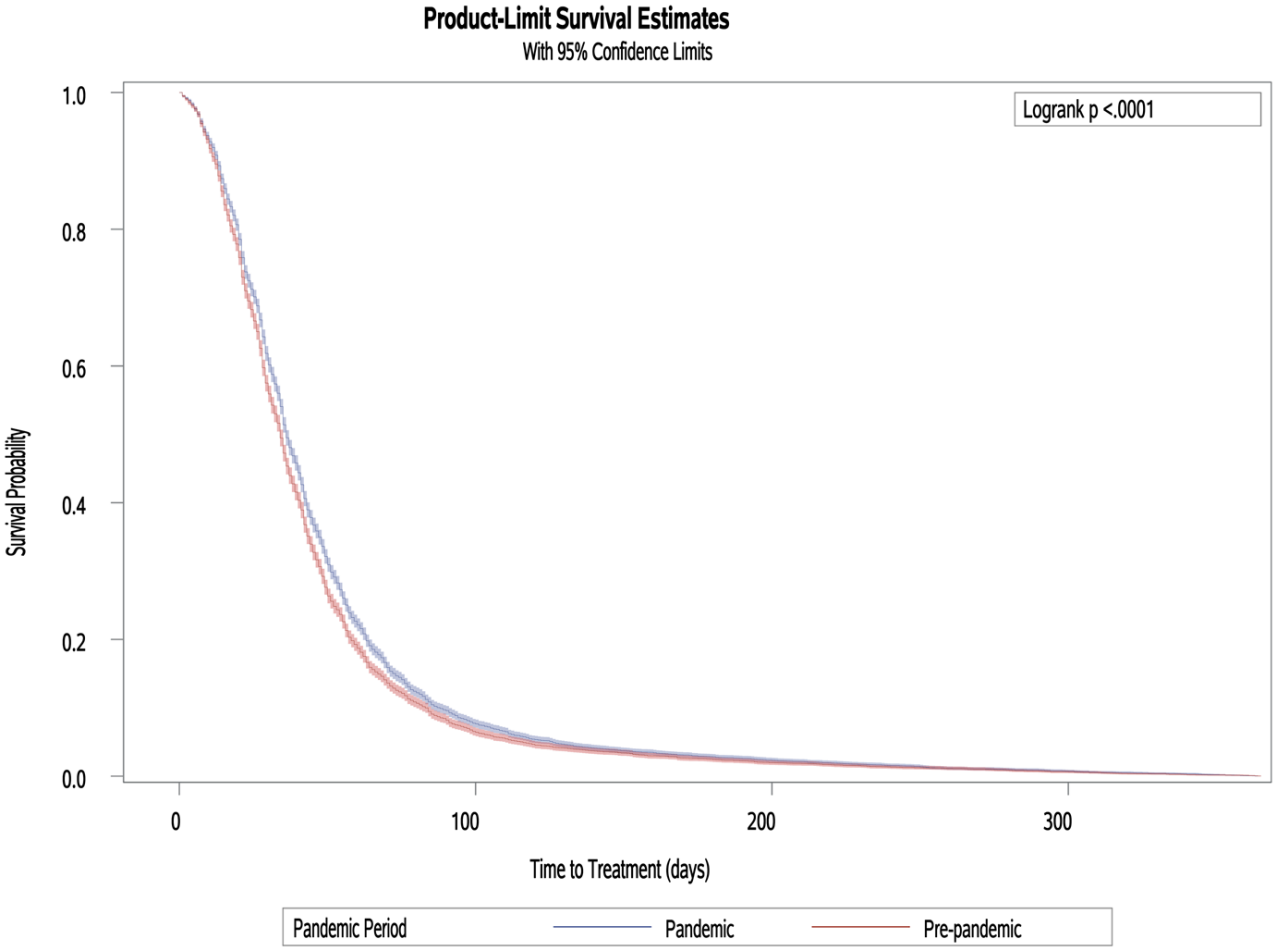
Kaplan–Meier curve demonstrating the significant difference in the average time to treatment, in days, for incident breast cancer by the study's COVID‐19 pandemic periods.

An unadjusted *t*‐test was performed to determine if there was a difference in the average time, in days, to treatment between those diagnosed during the pre‐pandemic and pandemic periods. We found a statistically significant difference in the average time (days) to treatment initiation between the two pandemic groups, *t*(14,284) = 3.75, *p* = 0.002. A greater average time to treatment was observed in those diagnosed during the pandemic period (47.69 days, 95% CI = 46.66–48.73) than in those diagnosed during the pre‐pandemic period (44.83 days, 95% CI = 43.75–45.90). A Kaplan–Meier survival analysis confirmed this result (Figure 2).

Table 2 details the results of the adjusted robust Poisson regression model for the binary outcome of timely treatment. After adjustment for age and beneficiary status, no significant difference in the likelihood of timely treatment was observed among those diagnosed during the pandemic period (0.99 aRR, 0.98–1.01 95% CI) compared to those diagnosed in the pre‐pandemic period. When compared to White women, no racial disparities in the likelihood of timely treatment were found. When compared to Senior Officers, which represents the highest socioeconomic strata in our cohort, there was also no observed difference in the likelihood of receiving timely treatment. In our final comparison of timely treatment by care setting, a higher likelihood of timely treatment was observed in the direct care setting (aRR = 1.04, 95% CI = 1.01–1.07) compared to the private sector care setting. Subset interaction analyses found no significant interaction between the pandemic periods and race, nor between pandemic periods and treatment care setting.

**TABLE 2:** Poisson regression results for the likelihood of timely treatment after incident breast cancer diagnosis.

	aRR (95% CI)
**Period of diagnosis**	
Pre‐pandemic (ref)	1
Pandemic	0.99 (0.98–1.01)
**Race**	
White (ref)	1
Black	0.98 (0.96–1.00)
Hispanic	0.98 (0.95–1.01)
Asian/Pacific Islander	0.98 (0.95–1.01)
American Indian/Alaskan Native	1.02 (0.97–1.03)
Other	0.98 (0.96–1.01)
**Rank**	
Junior enlisted	0.92 (0.81–1.04)
Senior enlisted	1.00 (0.98–1.02)
Junior officer	0.99 (0.96–1.02)
Senior officer (ref)	1
Warrant officer	0.99 (0.95–1.03)
Other	1.00 (0.93–1.07)
**Treatment care setting**	
Direct care	1.04 (1.01–1.07)^a^
Private sector care (ref)	1

*Note:* Models adjusted by patient age and beneficiary status. RWEE method was used to impute for missing race data.

Abbreviations: aRR, adjusted risk ratio; CI, confidence interval.

^a^ Indicates statistical significance, *p* < 0.05.

## 4 Discussion

In this study, we aimed to determine if the COVID‐19 pandemic impacted time to treatment and to evaluate racial disparities related to the timely initiation of treatment among women diagnosed with breast cancer in the MHS. Between the pre‐pandemic and pandemic periods, there was a significant difference in the average number of days to treatment, with a higher average observed in the pandemic period; however, these averages did not exceed the threshold definitions of timeliness per treatment type (chemotherapy, radiation, and surgery), and there was no difference observed in the receipt of timely treatment between the two periods. Additionally, racial and socioeconomic disparities in the receipt of timely treatment for incident breast cancer were found to be mitigated in the MHS during FY 2018–2022. These findings are in opposition to current literature [10, 11, 15, 16] and indicate equity in the receipt of treatment among female beneficiaries with breast cancer in the MHS.

COVID‐19 exacerbated existing challenges with access to care in the MHS [17]. DeGroff et al. [16] demonstrated severely decreased national breast cancer screening rates during the COVID‐19 pandemic, as high as an 89% decrease in May 2020 compared to the same month in the previous 5 years [16]. Breast cancer screening in the MHS dropped 74% in the early pandemic period and 22% in the late pandemic period, compared with the pre‐pandemic period [15]. Considering the worldwide impacts and delayed diagnosis of breast cancer due to COVID‐19, we expected to see a similar decrease in the percentage of women receiving timely treatment for breast cancer. However, this study found no difference in the receipt of timely treatment for incident breast cancer between the pre‐pandemic and pandemic periods. This could be that the MHS was able to keep up throughput during the pandemic from the decreased patient load from significantly lower screening rates. Despite challenges with access to care and a global pandemic, the MHS was able to provide timely treatment for incident breast cancer.

In the examination of racial and socioeconomic disparities in the receipt of timely treatment for incident breast cancer, this study's findings are in opposition to the literature and no significant differences were observed between racial‐ethnic and socioeconomic groups. Prior research of the MHS from 2014 to 2018 found that patients associated with a senior officer rank were more likely to receive timely initial treatment compared to patients associated with a junior enlisted rank [10]. Additionally, [11] concluded that race and socioeconomic factors were associated with decreased breast cancer screening rates during the COVID‐19 pandemic [11]. Our study's findings demonstrate health equity in the receipt of timely treatment among racial‐ethnic and socioeconomic groups from a broad perspective; however, further investigation is needed to determine if these findings persist when examined by treatment type and cancer staging.

### 4.1 Strengths and Limitations

The strengths of this study include the access and use of big health care data from a comprehensive health care system, and our cohort comes from a U.S. population that is nationally representative of the U.S. working age population [18, 19, 20]. Those eligible to use the MHS include active duty service members of all branches, retirees from active military service, guard and reserve personnel, and their dependents. This wide range of beneficiaries is diverse in age, race, rank (used as a proxy of socioeconomic status) [18, 21], and morbidity, and allowed for meaningful demographic analysis. Breast cancer in the general population translates easily to the MHS population, and extensive data was available for analysis.

There are also several limitations to this study. Firstly, there is the reliance on secondary health care claims data which carries the potential to underestimate disease and treatment due to coding errors. Secondly, our analysis focused on differences in the time from initial diagnosis to initial treatment by pandemic period, and whether or not the initial treatment was timely based on treatment type guidelines. This approach does not assess patterns or seasonal variability in treatment and represents an opportunity for further research. Another potential issue with the study design is that the pandemic period is longer than the pre‐pandemic period. This does not affect the measured outcomes of time to treatment or timely treatment; however, it does allow for more people to be included in the pandemic period. Fourthly, we found the cohort was largely missing race data (approximately 50%). Pattern analysis indicates race is missing at random (MAR) by beneficiary status (Table S2). This is largely due to a mandatory requirement of only active duty personnel to report their race and reporting of race as optional for all other MHS beneficiaries. We attempted to mitigate the impact of missing data by imputing race using the RWEE method, which has been found to perform better than complete case analysis under MAR conditions and introduce limited bias equivalent to multiple imputation [14]. Additionally, adjusted regression analyses with missing race included were performed, and the overall findings were consistent by pandemic period and across all racial groups (Table S3). Fifthly, the external validity or generalizability of the study is limited. This study lacked access to pathologic and clinical details such as tumor stage, grade, hormone receptor status, and histologic subtype, which are factors known to differ between racial‐ethnic and SES groups and thus may affect the analysis on time to treatment by demographic variables. These results are also only comparable to the MHS and other comprehensive health care systems. Additionally, active duty military account for 1% of the population; though individuals with any beneficiary status were included, the active duty population may experience unique environmental exposures compared to the general civilian population. And lastly, of the 16,225 women with an incident breast cancer diagnosis, 3070 did not receive treatment within 365 days of initial diagnosis. In a comparison of their demographics, this study found all characteristics except race to have statistically significant differences (Table S4). Continued investigation of these women is an opportunity for further research, such as understanding why these women did not receive treatment, if they were lost to follow‐up, or investigating if the experience of the untreated women vs. treated women was different within the MHS and if this played a role in their decision to receive care, and their mortality status.

## 5 Conclusion

This study observed no difference in the likihood of receiving timely treatment in the MHS between the pre‐pandemic and pandemic periods, despite the COVID‐19 pandemic and compounding access to care challenges. This study also found no racial or socioeconomic disparities in the receipt of timely treatment for incident breast cancer during fiscal years 2018–2022.

## Author Contributions

**Melody F. Mullin:** conceptualization, writing – original draft, writing – review and editing, validation, methodology. **Amanda Banaag:** conceptualization, writing – original draft, writing – review and editing, validation, formal analysis, data curation, visualization, methodology. **Christian Coles:** supervision, writing – review and editing, project administration, methodology, validation. **Yvonee Eaglehouse:** writing – review and editing, methodology. **Kangmin Zhu:** writing – review and editing, methodology. **Tracey P. Koehlmoos:** conceptualization, funding acquisition, supervision, project administration, methodology, validation, writing – review and editing.

## Ethics Statement

This study protocol was reviewed by the Uniformed Services University Institutional Review Board and received an exempt determination under the provision of DoD regulation 32 CFR 219.104(d)(4) and an approved waiver from obtaining informed consent under DOD 6025.18‐R, C7.9.2.2.

## Conflicts of Interest

The authors declare no conflicts of interest.

## Supporting information


**Table S1:** Sensitivity analysis results from the adjusted Poisson regression with patients diagnosed between January and March 2020 excluded.
**Table S2:** Pattern of missing race by patient demographics and pandemic period of diagnosis.
**Table S3:** Comparison of adjusted Poisson regression modeling with weighted race and missing race.
**Table S4:** Characteristics of patients without treatment for incident breast cancer, *N* = 3070.

## Data Availability

The data that support the findings of this study are available from the United States Defense Health Agency. Restrictions apply to the availability of these data, which were used under Federal Data User Agreements for the current study, and so are not publicly available.
