# Supplementary material for: Impact of COVID‐19 on Time to Treat Breast Cancer and Racial Disparities Among Women in the Military Health System From FY2018‐2022
Source: Cancer Med. 2025 Oct 6;14(19):e71292. doi: 10.1002/cam4.71292 (PMC12498276; doi:10.1002/cam4.71292)
Supplement: Supplementary file 1 — Table S1: Sensitivity analysis results from the adjusted Poisson regression with patients diagnosed between January and March 2020 excluded. Table S2: Pattern of missing race by patient demographics and pandemic period of diagnosis. Table S3: Comparison of adjusted Poisson regression modeling with weighted race and missing race. Table S4: Characteristics of patients without treatment for incident breast cancer, N = 3070. [file CAM4-14-e71292-s001.docx]

**Table S1. Sensitivity analysis results from the adjusted Poisson regression with patients diagnosed between January and March 2020 excluded**

|  | **aRR (95% CI)** |
| --- | --- |
| **Period of Diagnosis** |  |
| Pre-pandemic (ref) | 1 |
| Pandemic | 0.99 (0.98 - 1.01) |
| **Race** |  |
| White (ref) | 1 |
| Black | 0.98 (0.96 - 1.00) |
| Hispanic | 0.98 (0.95 – 1.01) |
| Asian/Pacific Islander | 0.98 (0.95 - 1.01) |
| American Indian/Alaskan Native | 1.01 (0.97 - 1.06) |
| Other | 0.98 (0.96 - 1.00) |
| **Rank** |  |
| Junior Enlisted | 0.91 (0.81 – 1.03) |
| Senior Enlisted | 1.00 (0.98 – 1.02) |
| Junior Officer | 0.99 (0.96 – 1.02) |
| Senior Officer (ref) | 1 |
| Warrant Officer | 0.99 (0.96 – 1.03) |
| Other | 1.00 (0.93 - 1.07) |
| **Treatment Care Setting** |  |
| Direct Care | 1.02 (1.01 – 1.04)* |
| Private Sector Care (ref) | 1 |

**Note:** Models are adjusted by patient age and beneficiary status. RWEE method was used to impute for missing race data.

**Abbreviations:** aRR = adjusted risk ratio; CI = confidence interval.

* Indicates statistical significance, p<0.05.

**Table S2. Pattern of Missing Race by Patient Demographics and Pandemic Period of Diagnosis**

|  | **Missing Race** | **No Missing Race** |
| --- | --- | --- |
| **Beneficiary Status** |  |  |
| Active Duty | 0 | 301 (4.19) |
| Dependent of Guard/Reserve | 667 (9.40) | 194 (2.70) |
| Dependent | 6376 (89.88) | 5370 (74.67) |
| Guard/Reserve | 0 | 226 (3.14) |
| Retiree | 13 (0.18) | 1021 (14.20) |
| Missing | 38 (0.54) | 80 (1.11) |
| **Rank** |  |  |
| Junior Enlisted | 259 (3.65) | 135 (1.88) |
| Senior Enlisted | 4749 (66.94) | 4945 (68.76) |
| Junior Officer | 718 (10.12) | 706 (9.82) |
| Senior Officer | 1107 (15.60) | 1090 (15.16) |
| Warrant Officer | 247 (3.48) | 243 (3.38) |
| Other/Missing | 14 (0.20) | 73 (1.01) |
| **Period of Diagnosis** |  |  |
| Pre-pandemic | 3359 (47.35) | 3188 (44.33) |
| Pandemic | 3735 (52.65) | 4004 (55.67) |

**Table S3. Comparison of adjusted Poisson regression modeling with weighted race and missing race**

|  | **Poisson Regression Model with Weighted Race**  **aRR (95% CI)** | **Poisson Regression Model with Missing Race**  **aRR (95% CI)** |
| --- | --- | --- |
| **Period of Diagnosis** |  |  |
| Pre-pandemic (ref) | 1 | 1 |
| Pandemic | 0.99 (0.98 - 1.01) | 1.01 (0.99 – 1.02) |
| **Race** |  |  |
| White (ref) | 1 | 1 |
| Black | 0.98 (0.96 - 1.00) | 0.99 (0.98 – 1.01) |
| Hispanic | 0.98 (0.95 – 1.01) | 0.98 (0.96 – 1.01) |
| Asian/Pacific Islander | 0.98 (0.95 - 1.01) | 0.99 (0.97 – 1.03) |
| American Indian/Alaskan Native | 1.01 (0.97 - 1.06) | 0.99 (0.92 – 1.08) |
| Other | 0.98 (0.96 - 1.00) | 0.99 (0.97 – 1.01) |
| Missing | - | 1.01 (0.99 – 1.02) |
| **Rank** |  |  |
| Junior Enlisted | 0.91 (0.81 – 1.03) | 0.98 (0.95 – 1.01) |
| Senior Enlisted | 1.00 (0.98 – 1.02) | 1.00 (0.99 – 1.01) |
| Junior Officer | 0.99 (0.96 – 1.02) | 1.00 (0.98 – 1.01) |
| Senior Officer (ref) | 1 | 1 |
| Warrant Officer | 0.99 (0.96 – 1.03) | 1.00 (0.98 – 1.03) |
| Other | 1.00 (0.93 - 1.07) | 1.03 (0.98 – 1.09) |
| **Treatment Care Setting** |  |  |
| Direct Care | 1.02 (1.01 – 1.04)* | 1.02 (1.01 – 1.03)* |
| Private Sector Care (ref) | 1 | 1 |

**Note:** Models are adjusted by patient age and beneficiary status. RWEE method was used to impute for missing race data.

**Abbreviations:** aRR = adjusted risk ratio; CI = confidence interval.

* Indicates statistical significance, p<0.05.

**Table S4. Characteristics of Patients without Treatment for Incident Breast Cancer, N=3,070**

|  | **Untreated Group, N=3,070**  **N (%)** | **Treated Group, N=14,286**  **N (%)** | **p-value** |
| --- | --- | --- | --- |
| **Mean age (st dev)** | 53.12 (8.21) | 52.30 (8.73) | <0.0001 |
| **Race** |  |  | 0.1717 |
| White | 777 (25.31) | 3914 (27.40) |  |
| Black | 335 (10.91) | 1453 (10.05) |  |
| Hispanic | 133 (4.33) | 566 (3.96) |  |
| Asian/Pacific Islander | 107 (3.49) | 468 (3.28) |  |
| American Indian/Alaskan Native/Other | 187 (6.09) | 808 (5.65) |  |
| Missing | 1531 (49.87) | 7094 (49.66) |  |
| **Beneficiary Status** |  |  | <0.0001 |
| Active Duty | 62 (2.02) | 301 (2.11) |  |
| Dependent of Guard/Reserve | 159 (5.18) | 861 (6.03) |  |
| Dependent | 22415 (78.66) | 11746 (82.22) |  |
| Guard/Reserve | 51 (1.66) | 226 (1.58) |  |
| Retiree | 280 (9.12) | 1034 (7.24) |  |
| Missing | 103 (3.36) | 118 (0.83) |  |
| **Rank** |  |  | <0.0001 |
| Junior Enlisted | 110 (3.58) | 394 (2.76) |  |
| Senior Enlisted | 2040 (66.45) | 9694 (67.86) |  |
| Junior Officer | 299 (9.74) | 1424 (9.97) |  |
| Senior Officer | 446 (14.53) | 2197 (15.38) |  |
| Warrant Officer | 123 (4.01) | 490 (3.43) |  |
| Other | 25 (0.81) | 44 (0.31) |  |
| Missing | 27 (0.88) | 43 (0.30) |  |
| **Period of Diagnosis** |  |  | 0.0034 |
| Pre-pandemic | 1318 (42.93) | 6547 (45.83) |  |
| Pandemic | 1752 (57.07) | 7739 (54.17) |  |

**Note:** Due to cell counts of 10 or fewer, American Indian/Alaska Native and ‘other’ race groups were combined to censor and protect the anonymity of patients.
